# Rumination Mediates the Relationship between Infant Temperament and Adolescent Depressive Symptoms

**DOI:** 10.1155/2011/487873

**Published:** 2010-09-01

**Authors:** Amy H. Mezulis, Heather A. Priess, Janet Shibley Hyde

**Affiliations:** ^1^Department of Clinical Psychology, Seattle Pacific University, 3307 3rd Avenue West Suite 107, Seattle, WA 98119, USA; ^2^Department of Psychology, University of Wisconsin–Madison, 1202 West Johnson Street, Madison, WI 53706, USA

## Abstract

This study examined prospective associations between negative emotionality, rumination, and depressive symptoms in a community sample of 301 youths (158 females) followed longitudinally from birth to adolescence. Mothers reported on youths' negative emotionality (NE) at age 1, and youths self-reported rumination at age 13 and depressive symptoms at ages 13 and 15. Linear regression analyses indicated that greater NE in infancy was associated with more depressive symptoms at age 15, even after controlling for child gender and depressive symptoms at age 13. Moreover, analyses indicated that rumination significantly mediated the association between infancy NE and age 15 depressive symptoms in the full sample. When analyzed separately by gender, however, rumination mediated the relationship between NE and depressive symptoms for girls but not for boys. The results confirm and extend previous findings on the association between affective and cognitive vulnerability factors in predicting depressive symptoms and the gender difference in depression in adolescence, and suggest that clinical interventions designed to reduce negative emotionality may be useful supplements to traditional cognitive interventions for reducing cognitive vulnerability to depression.

## 1. Introduction

Adolescent depression is a major mental health problem. Depression increases in the transition to adolescence, such that while fewer than 6% of youth under age 11 will experience a depressive episode, nearly 20% of youth will experience a depressive episode by age 18 [1, 2]. In addition, up to 65% of adolescents report subclinical depressive symptoms at any given time, and extensive research has demonstrated that both mild-to-moderate depressive symptoms and diagnosable depressive episodes predict greater academic and interpersonal problems, substance use, and depressive episodes in adulthood [3, 4]. Adolescent depression also confers risk for future depression, with nearly 70% of adolescents experiencing another episode within five years [5]. Within adolescence, the early to middle adolescent period from ages 11 to 15 is of particular salience to depression researchers. During this time, depression rates surge for all youth and a marked gender difference emerges such that by age 15 girls are twice as likely as boys to become depressed [2].

Extensive research on the etiology of adolescent depression has demonstrated multiple vulnerability factors contributing to the rise in depressive symptoms as well as the emergence of the gender difference in depression during this developmental period. Both affective (e.g., temperament) and cognitive (e.g., rumination, cognitive style) factors have been found to confer vulnerability to adolescent depression. In their ABC Model of depression in adolescence, Hyde et al. [6] integrated affective and cognitive models of adolescent depression by hypothesizing that the specific temperamental trait of negative emotionality contributes to the development of maladaptive cognitive responses, such as rumination, that become habitual across the adolescent transition and subsequently confer vulnerability to depression. The current study examines this hypothesis longitudinally in a sample of community youth followed from infancy into adolescence.

Temperament is conceptualized as biologically-based, relatively stable individual differences in emotional, behavioral, and attentional reactivity and regulation [7]. These individual differences are hypothesized to be present early in infancy and childhood, and relatively stable across the lifespan. Negative emotionality (NE) is defined as a constellation of temperamental characteristics including high frequency and intensity of negative affective states such as fear, frustration, and distress. In infancy, children high in NE tend to display strong startle responses to new or aversive stimuli as well as high distress to novel or frustrating situations. Later in childhood, youth high in NE tend to dislike or avoid novel situations, become highly distressed in novel or frustrating situations, and display negative emotions such as fear, distress, and frustration more frequently and/or intensely than other children [8, 9]. Numerous studies have demonstrated an association between NE and depression among adults, adolescents, and children [10–13]. Specifically, NE has been demonstrated to be both concurrently associated with and prospectively predictive of depression in adolescence [10, 14]. 

The cognitive model of depression suggests that individual differences in cognitive responses to negative events may predispose individuals to becoming depressed when faced with such events. One such cognitive vulnerability is a ruminative response style [15, 16]. Rumination may be broadly defined as a passive and perseverative attentional focus on negative stimuli, including sad, depressed, or negative emotions, stressful events, and self-critical or otherwise negative thoughts. Nolen-Hoeksema originally defined rumination broadly as “repetitively focusing on the fact that one is depressed; on one's symptoms of depression; and on the causes, meanings, and consequences of depressive symptoms” [15, page 569]. In recent years, researchers have identified many subtypes of rumination differentiated primarily by the content upon which an individual is ruminating. One such subtype of rumination has been termed *depressive rumination*. Depressive rumination is defined as a passive and perseverative focus on negative emotions such as sadness and depressed mood [17–20]. Depressive rumination reflects an involuntary coping response in which one's attention following a stressor is “directed passively and perseveratively toward the negative emotions elicited by the stressor” [21, page 977]. As such, depressive rumination is essentially emotion-focused and can be differentiated from other rumination subtypes, including brooding, which is ruminative focus on negative or self-critical thoughts [22] and from rumination about other negative emotions such as anger [18, 23]. 

Rumination on sadness and depressed feelings is believed to prolong and exacerbate depressed mood by increasing the salience of the negative emotions being attended to. Not surprisingly, then, depressive rumination has been demonstrated to prospectively predict both the onset and duration of depression among adolescents [24, 25]. However, we understand less about the factors contributing to individual differences in depressive rumination. Given the emotion-focus of depressive rumination, individual differences in negative emotionality may be associated with individual differences in the tendency to ruminate on those negative emotions. 

In their ABC Model of depression in adolescence, Hyde et al. [6] integrated affective and cognitive models of depression by suggesting that youth high in negative emotionality would, over time, develop more negative cognitive responses to stressful events. This hypothesis integrates basic research linking affective reactions to stressful events to the cognitive processing of those events, and further frames the integration within the developmental trajectory of adolescent depression. Weiner [26] noted that affective processing of negative events precedes higher-order cognitive processing of the event, such as making attributions about causality. Several studies have demonstrated that affective responses to stressful events subsequently affect cognitive processing of those events. For example, high negative affect is associated with greater subjective appraisal of ambiguous or novel events as stressful [27]. High negative affect is also associated with greater interpretation of events as catastrophic, greater attention to the negative event, greater self-focus, and more negative expectancies [28–30]. Over time, this pattern of more negative and depressogenic cognitive responding may become habitual and consolidate into a trait of cognitive vulnerability to depression such as rumination. Several researchers have suggested that cognitive vulnerabilities to depression emerge and consolidate in early to middle adolescence, a timing that is consistent with the increase in depressive symptoms among youth [31, 32].

This hypothesized developmental link between negative emotionality and rumination suggests that the predictive relationship between negative emotionality and depression may be mediated in part by rumination. In recent years, a handful of studies have examined this hypothesis empirically. Feldner et al. reported that, among adults, negative emotionality was significantly correlated with rumination [33]. A similar correlation has been observed among adolescents as well [34]. Verstraeten et al. tested the full mediation model among adolescents and found that rumination did significantly mediate the relationship between negative emotionality and depression, but only when constructs were analyzed concurrently rather than prospectively [35]. To the best of our knowledge, only one study has examined the relationship between negative emotionality, rumination, and depression prospectively among adolescents. Mezulis et al. recently found that the prospective relationship between negative emotionality in early adolescence (age 12) and later depressive symptoms (age 15) was mediated by rumination at age 14, even after controlling for depressive symptoms at ages 12 and 14 [36]. A limitation of this study, though, is that all measures were self-reported by the youth in adolescence, leaving unanswered the question of whether indices of temperament early in life are associated with the development of rumination and subsequent depression in adolescence.

Finally, it is as yet unknown whether prospective relationships among negative emotionality, rumination, and depression may contribute to our understanding of the emergent gender difference in depression in adolescence. Although many studies identify a gender difference in rumination that has been found to partially mediate the gender difference in depression in adolescence (see [6] for a review), most studies fail to demonstrate a significant gender difference in negative emotionality in infancy or childhood [37]. It is possible that negative emotionality may contribute to the development of rumination amongst girls but not boys, perhaps because of how coping responses to negative mood are socialized differently among boys and girls [38]. Thus, we hypothesized that the prospective relationship between negative emotionality and rumination may be stronger for girls than boys.

**Figure 1:**
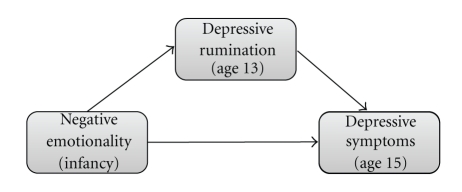
Conceptual model.

The current study extends the extant literature by examining rumination as a mediator of the relationship between negative emotionality in infancy and depressive symptoms in adolescence in a prospective study of community youth followed longitudinally from infancy to age 15. The conceptual model is presented in Figure 1. Specifically, we hypothesized that:

(1) negative emotionality in infancy would predict depressive symptoms in mid-adolescence (age 15);
(2) the relationship between infant negative emotionality and adolescent depressive symptoms at age 15 would be mediated by rumination at age 13, even after controlling for depressive symptoms at age 13;
(3) the relationship between infant negative emotionality and rumination at age 13 would be stronger for girls than boys.

## 2. Method

### 2.1. Participants

Participants were 301 adolescents (158 female) in the United States who have been part of the longitudinal Wisconsin Study of Families and Work since birth (formerly named the Wisconsin Maternity Leave and Health Project; [39]). Participants were originally recruited from the Madison and Milwaukee, Wisconsin areas and currently reside in a range of communities, including a large Midwestern city, a small Midwestern city, several small towns, and rural areas. Of participants in the present study, 90.0% were White, 4.0% American Indian/Alaskan, 3.0% African American, 1.7% Asian/Pacific Islander, 1.0% Hispanic, and 0.3% were members of another group. Participants are ethnically representative of the communities from which they were recruited, and their families are socioeconomically similar to families in the United States. 

Data were collected at age one and during the summer following grades seven (mean age = 13.52,  SD = 0.33; summers of 2004 and 2005) and nine (M = 15.50, SD = 0.33; summers of 2006 and 2007). The present study includes participants who completed all measures used in the study. Participants who remained in the study at adolescence did not differ from those who discontinued participation prior to adolescence in terms of race/ethnicity, family income, or parents' depressive symptoms. 

### 2.2. Procedure

When the participating children were 12 months of age, their mothers completed a written questionnaire to assess the children's temperament. At ages 13 and 15, participants completed a number of questionnaires administered on a laptop computer during in-home visits. These questionnaires included measures of depressive symptoms in the past two weeks and rumination tendencies.

### 2.3. Measures

#### 2.3.1. Negative Emotionality

Negative emotionality was measured using the distress to limitations (anger), distress to novelty (fear), and startle subscales of the Infant Behavior Questionnaire (IBQ; [40]). The 39 items of these subscales asked mothers to rate their children's responses to specific behaviors (e.g., child became upset when having face washed) in the past two weeks on a seven-point scale ranging from 1 (*never*) to 7 (*always*). Negative emotionality was calculated by averaging the mean scores on each of the three subscales. Rothbart reported internal consistencies for children 12 months of age as.78 for distress to limitations and.81 for distress to novelty. In the present study, internal consistency was.84 for distress to limitations,.74 for both distress to novelty and startle, and.84 for the combined set of items.

#### 2.3.2. Rumination

Depressive rumination was assessed at age 13 using a short form of the Ruminative Response Scale (RRS) of the Response Style Questionnaire (RSQ; [41]). The original RRS includes 22 items in which respondents are asked how often they engage in ruminative responses when they feel sad or down, with responses rated on a 4-point Likert scale from 1 (*almost never*) to 4 (*almost always*). In the current study, we used five items specifically assessing rumination about negative affect. Items include “When I feel sad or down, I think about how alone I feel” and “When I feel sad or down, I think about how hard it is to concentrate”. The five items utilized were selected based upon consultation with Nolen-Hoeksema at the time of study design (personal communication, 2001) as representing a selection of rumination items that excluded automatic negative thoughts and emphasized instead rumination about sad, depressed, or down affect. The exclusion of items that include negative automatic thoughts is preferable because it creates a purer measure of depressive rumination that is focused on the affective component of depressive symptoms, rather than the cognitive component. The full RRS has been used with adolescents in several prior studies [42, 43]; in the present study, internal consistency was .73 for the depressive rumination subscale.

#### 2.3.3. Depressive Symptoms

Depressive symptoms were assessed at ages 13 and 15 with the Children's Depression Inventory (CDI; [43]). The CDI includes 27 items assessing common affective, behavioral, and cognitive symptoms of depression. For each of the 27 items, adolescents were asked to pick which of three statements best described them in the past two weeks. The three statements represent differing levels of symptom severity; for example, youth select one of the following three statements: “I was sad once in a while,” “I was sad many times,” or “I was sad all the time.” Each item is scored 0, 1, or 2, and answers to individual items were summed, such that a CDI score could range from 0 to 54. The CDI has demonstrated good internal consistency (typically .71 to .89) and adequate test-retest reliability (typically .72 to .87) [44–46]. In the present study, internal consistency was .83 at age 13 and .86 at age 15.

### 2.4. Data-Analytic Technique

Analyses were conducted in SPSS as a series of regression models (and in the case of the indirect effect, confidence intervals) using the macro command set developed by Preacher and Hayes [47] to test mediation models that include covariates. A mediation model suggests that the relationship between the predictor variable (here, negative emotionality) and the outcome variable (here, depressive symptoms at age 15) is partially or completely accounted for by some mediating variable (here, rumination at age 13). With the mediator in the model, the predictor and outcome variables are not expected to be directly related, but rather *indirectly* related through the effect of the predictor variable on the mediator, and then the mediator variable on the outcome variable. 

Common tests of indirect effects in mediation models, such as the Sobel test, assume that the sampling distribution of an indirect effect is normally distributed; however, this assumption typically holds only for quite large sample sizes. To avoid violating this assumption, the procedure developed by Preacher and Hayes uses a nonparametric approach that does not require multivariate normality to explicitly test the indirect effect. Specifically, this procedure employs a bootstrap method, in which the original data are sampled (with replacement) 5000 times. The indirect effect coefficients generated from these 5000 samples are then ordered numerically. The low and high values that cap the middle 95% of the results represent the bounds of a 95% confidence interval. If zero is not within this 95% confidence interval, then there is evidence of an indirect effect between the predictor variable (e.g., negative emotionality) and outcome variable (e.g., depressive symptoms) at the standard Type I error rate of *α* = .05.

We examined the hypothesized mediator model three times: once for the entire sample, and then separately for boys and girls.

## 3. Results

### 3.1. Descriptive Statistics

Table 1 displays descriptive statistics for overall negative emotionality, rumination, and depressive symptoms, separately by gender.  Table 2 displays correlations between negative emotionality, rumination, and depressive symptoms, again separately by gender. As seen in Table 2, the patterns of correlations varied markedly for girls and boys. Therefore, analyses were computed first for the entire sample and then separately for girls and boys.

### 3.2. Temperament, Depressive Rumination, and Depressive Symptoms

The present study examined a mediation model in which depressive rumination at age 13 was hypothesized to mediate the relationship between negative emotionality in infancy and depressive symptoms at age 15. Regression equations tested each pathway depicted in Figure 1. In addition, each equation controlled for prior depressive symptoms at age 13 and gender. Depressive symptom scores were log transformed prior to analysis to account for their skewed distribution. 

Results supported our hypothesized mediation model. The effect of negative emotionality on depressive symptoms at age 15 was significant, *b* = .09, *t*(300) = 2.23, *P* = .03. Additionally, as expected, the path from negative emotionality to rumination was significant; participants who were higher in negative emotionality during infancy reported greater tendencies to ruminate as adolescents, *b* = .14, *t*(300) = 2.57, *P* = .01. Similarly, the path from rumination to depressive symptoms was significant; adolescents who reported more rumination at age 13 had more depressive symptoms at age 15, even after controlling for earlier symptoms, *b* = .12, *t*(300) = 2.99, *P* = .003. With rumination in the model, the direct effect of negative emotionality on depressive symptoms at age 15 was nonsignificant, *b* = .02, *t*(300) = .61, *P* = .54. Additionally, the confidence interval for the effect of the indirect pathway via rumination did not include “0” (.004 to.037), indicating a significant mediated pathway. The overall mediation model was significant, *F*(4,296) = 34.14, *P* < .001, and accounted for approximately 32% of the variance in depressive symptoms (*R* ^2^ = .32, adjusted *R* ^2^ = .31). Thus, our model of the relationship between negative emotionality, rumination, and depressive symptoms was supported by the data.

#### 3.2.1. Temperament, Rumination, and Depressive Symptoms among Girls

Given the marked differences in the correlations among variables for boys and girls in our sample, we also examined the hypothesized mediation model separately by child gender to determine if the relationships among variables were comparable across gender. The overall model for girls was significant, *F*(3,154) = 24.61, *P* < .001, and explained 32% of the variance in depressive symptoms (*R* ^2^ = .32, adjusted *R* ^2^ = .31). The effect of infant negative emotionality on depressive symptoms at age 15 failed to reach statistical significance, likely as a result of the reduced sample size (*b* = .08, *t*(157) = 1.48, *P* = .14). However, the path from negative emotionality to rumination was significant; as expected, girls who were higher in negative emotionality during infancy reported greater tendencies to ruminate as adolescents, *b* = .16, *t*(157) = 2.09, *P* = .04. Similarly, the path from rumination to depressive symptoms was significant; girls who reported more rumination at age 13 had more depressive symptoms at age 15, even after controlling for symptoms at age 13, *b* = .16, *t*(157) = 2.86, *P* = .005. Finally, the confidence interval for the indirect pathway via rumination did not include “0” (.004 to .061), indicating that rumination significantly mediated the relationship between negative emotionality and depressive symptoms among girls. 

#### 3.2.2. Temperament, Rumination, and Depressive Symptoms among Boys

The overall model for boys was significant, *F*(3,139) = 16.04, *P* < .001, and explained 26% of the variance in depressive symptoms (*R* ^2^ = .26, adjusted *R* ^2^ = .24). The effect of infant negative emotionality on depressive symptoms at age 15 was comparable to that observed among girls and also failed to reach statistical significance, also likely as a result of the reduced sample size (*b* = .08, *t*(142) = 1.32, *P* = .19). However, in contrast to girls, the effect of negative emotionality on rumination was not significant; boys who were higher in negative emotionality during infancy did not report greater tendencies to ruminate as adolescents, *b* = .09, *t*(142) = 1.11, *P* = .27. Furthermore, the effect of rumination on depressive symptoms was also not significant; rumination at age 13 did not predict depressive symptoms at age 15 among boys, *b* = .08, *t*(142) = 1.36, *P* = .18. Finally, the confidence interval for the indirect pathway via rumination did include “0” (−.005 to .030), indicating that rumination did not significantly mediate the relationship between negative emotionality and depressive symptoms among boys.

## 4. Discussion

This study examined the relationship between temperament, rumination, and depressive symptoms prospectively in a community sample of youth followed from infancy to age 15. The primary purpose of the study was to empirically test one of the integrative hypotheses of the ABC Model of adolescent depression which asserts that affective vulnerability to depression, that is, temperament, contributes to the development of cognitive vulnerability to depression, that is, rumination [6]. This hypothesis suggests that individuals who are temperamentally predisposed to respond to stressful events with intense and prolonged negative affect will subsequently allocate more attentional resources to those events, and that this pattern of affective-cognitive processing of stressful events will, over time, consolidate into the stable cognitive vulnerability of rumination. The present study extends prior examinations of rumination as a mediator of the relationship between temperament and depression by examining infant temperament as it predicts later rumination and depressive symptoms.

### 4.1. Infant Negative Emotionality and Adolescent Depression

Numerous studies have examined the temperamental construct of negative emotionality as it predicts depressive symptoms and disorders [7, 13]. However, only a handful of studies have examined the predictive relationship between infant or early childhood temperament on depression in adolescence. Moffitt et al. [48] reported that behavioral inhibition, one component of negative emotionality, at age 3 prospectively predicted depression diagnoses by age 21. Similarly, Lonigan et al. found that childhood negative emotionality at age 9 predicted depressive symptoms at age 16 [49]. Consistent with these prior studies, we similarly found that greater negative emotionality in infancy was significantly associated with greater depressive symptoms in adolescence. 

### 4.2. Linking Affective and Cognitive Processes to Adolescent Depression

The primary purpose of this study, however, was to examine the mechanism by which early individual differences in negative emotionality develop into later depressive symptoms. In adolescence, we know that individual differences in affective and cognitive responses to stressful events may differentiate individuals for whom mood disturbances are transient from individuals for whom that mood disturbance persists and develops into a depressive response. Teasdale's differential activation hypothesis [30] suggests that some individuals are more likely to respond to negative affect with the activation of negative thoughts and rumination. Teasdale labeled this individual difference in the extent to which negative cognitive processing is elicited by negative affect as *cognitive reactivity, *and suggested that for these cognitively reactive individuals, a vicious cycle between negative affect and negative thinking will ensue that eventually leads to depression. The ABC Model presents a theoretically consistent hypothesis that emphasizes the *developmental* relationship between negative affect and cognitive processing, suggesting that it is early individual differences in affective responding to stress that set the developmental stage for this vicious cycle of affective and cognitive processing to ensue. We hypothesized that children who are temperamentally high in negative emotionality will become adolescents who are high in cognitive vulnerability, including rumination.

Only a handful of studies have examined whether individual differences in negative emotionality predict individual differences in cognitive vulnerability to depression. Several studies have examined the relationship between neuroticism, rumination, and depression among adults [50–55]. Neuroticism is a personality trait which is similar to, but more broad than, the temperamental construct of negative emotionality; neuroticism is associated with high negative emotionality as well as high stress sensitivity and worry [56].

Few studies have examined the relationship between the specific construct of negative emotionality and rumination. Our findings contribute to a small but growing body of literature suggesting that negative emotionality is an important contributor to rumination among adolescents. Our findings are consistent with those of Chang [34] and Verstraeten et al. [35] demonstrating a significant association between these constructs, and providing further evidence that the relationship between temperament and depression may be mediated by cognitive processes.

Interestingly, although the mediation model was supported for the entire sample, follow-up analyses by child gender suggested that the prospective association between infant negative emotionality and adolescent depressive symptoms may be mediated by rumination among girls but not among boys. This finding suggests, consistent with the ABC Model of the gender difference in adolescence depression, that there may be multiple processes contributing to the gender difference in depression. In our sample, girls and boys did not differ on mother-reported infant negative emotionality, a finding consistent with a recent meta-analysis examining gender differences in temperament [37]. However, boys and girls did differ on rumination in early adolescence, with girls reporting greater rumination than boys. Our findings suggest that early negative emotionality may contribute to later ruminative tendencies among girls but not boys. It is interesting to speculate on this gender divergence; one possible explanation may be how parents respond to displays of negative emotionality differently for sons and daughters. Recent research has suggested that mothers may be more likely to direct attention to and encourage discussion of negative emotions, particularly fear, distress, and sadness, when interacting with their daughters than with their sons, and that this gender difference in parenting style may contribute to the gender difference in rumination [38]. 

It is also important to note that negative emotionality is only one of multiple constructs of temperament associated with vulnerability to depression. Prior research has also implicated low positive emotionality in the etiology of depression [12], although that relationship is not hypothesized to be mediated by cognitive vulnerability [6]. Recent research has also suggested that the relationship between negative emotionality and cognitive vulnerability may itself by moderated by other regulatory components of temperament. For example, effortful control is another feature of temperament conceptualized as “the ability to inhibit a dominant response to perform a subdominant response” [7, page 137], and thus is a self-regulatory process that requires effortful or voluntary control of both attention and behavior to modulate emotional experience and expression [57]. Some have found that effortful control may moderate the relationship between the affective reactivity of negative emotionality and rumination [35]. Although the present study did not include a measure of effortful control with which to examine this hypothesis, it would be an interesting elaboration for future prospective studies.

### 4.3. Clinical Implications

Adolescent-onset depression is associated with both concurrent deficits in adaptive functioning and prospective risk for future depressive episodes. The vast majority of youth experiencing depression in adolescence will have another episode within five years [5]. Unfortunately, treatments for adolescent depression lag behind those for other disorders and more than half of youth fail to respond to currently available interventions [58]. The majority of current depression interventions emphasize techniques designed to reduce or eliminate depressogenic cognitive processes such as rumination. Continued evidence suggesting that individual differences in negative emotionality significantly contribute to individual differences in rumination suggest that interventions designed to reduce individuals' negative affect may be helpful in treating or preventing depression as well. Relaxation training may be effective in attenuating youths' automatic and intense negative affective responses, and a growing body of research suggests that mindfulness-based interventions may improve emotion regulation through improving the individual's ability to respond to stressful situations reflectively rather than automatically. Mindfulness has been shown to be effective in treating depression, possibly through its effects in decreasing rumination [59, 60]. Kabat-Zinn [61] also suggested that the slow and deep breathing taught in mindfulness training may reduce physiological reactivity and the subjective emotional and physiological feelings of distress. Although few studies have examined the effects of mindfulness on affective responding, Goldin and Gross [62] recently reported that individuals with social anxiety reported less negative affect during a breath-focused mindfulness task compared to a distraction-focused task. Similarly, Charbonneau and Mezulis [63] recently reported that among college females high in negative emotionality, a brief intervention teaching emotion regulation strategies such as deep breathing and progressive muscle relaxation strategy was effective in reducing rumination. In summary, a greater understanding of the relationship between affective and cognitive vulnerability to depression may suggest that clinical interventions targeting negative emotionality or emotional reactivity may be effective in reducing cognitive vulnerability to depression as well.

### 4.4. Limitations and Future Research

While our study demonstrates links between temperament, rumination, and depressive symptoms, several limitations should be noted. First, the study examined rumination narrowly defined as perseverative attention on negative emotions. Conceptually, the depressive rumination subtype of rumination is most logically linked with negative emotionality, as the strong negative emotions experienced by individuals high in this temperamental feature may particularly elicit the attentional focus on negative emotions that comprises depressive rumination. However, future research may want to consider the relationship between negative emotionality and other forms of rumination. Of particular interest may be brooding, which is defined as rumination on negative or self-critical thoughts. Recent evidence specifically links the rumination dimension of brooding with depression among adolescents and may be one specific facet of the cognitive reactions to stress elicited by negative emotionality [64, 65]. Second, our sample was a community sample of predominantly Caucasian youth. The relationships among our constructs may be different among high-risk or clinical samples. Finally, we note that the pathway from negative emotionality to depression, mediated by rumination, demonstrated in the current sample is but one of multiple developmental trajectories implicated in the etiology of adolescent depression. As extensively reviewed in other studies, there are multiple temperamental and cognitive factors that contribute to adolescent depression both within and across individuals. Low positive emotionality is another temperamental factor implicated in the etiology of depression, as well as other cognitive vulnerability factors such as negative cognitive style (as reviewed in [6]). Thus, the negative emotionality-rumination-depression link examined here is undoubtedly but one pathway to adolescent depression.

Despite these limitations, our findings continue to implicate temperament in the development of cognitive vulnerability to depression. These findings contribute to our ability to identify at-risk individuals as well as design interventions targeting both affective and cognitive processes in adolescent depression.

## Figures and Tables

**Table 1:** Descriptive statistics for overall negative emotionality, rumination, and depressive symptoms, by gender.

	Girls		Boys		Comparison	
Variable	*M*	*SD*	*M*	*SD*	* t*	*P*
NE (infancy)	2.94	.58	2.84	.56	1.46	.146
Rumination (13)	2.02	.55	1.77	.53	3.91	.000
Depression (13)	4.89	5.21	3.79	4.18	2.01	.046
Depression (15)	5.74	5.82	3.84	4.95	3.03	.003

**Table 2:** Correlation matrix for overall negative emotionality, rumination, depressive symptoms, and gender.

	1	2	3	4
(1) NE (infancy)		.09	.08	.14
(2) Rumination (13)	.17*		.23**	.15
(3) Depression (13)	.10	.52**		.45**
(4) Depression (15)	.07	.45**	.62**	

*Note*. Correlations significant at *P* < .05 denoted by *. Correlations significant at *P* < .01 denoted by **. Correlations are above the diagonal for boys and below the diagonal for girls.
